# Barriers impacting the POINT pragmatic trial: the unavoidable overlap between research and intervention procedures in “real-world” research

**DOI:** 10.1186/s13063-021-05065-3

**Published:** 2021-02-04

**Authors:** Allyson L. Dir, Dennis P. Watson, Matthew Zhiss, Lisa Taylor, Bethany C. Bray, Alan McGuire

**Affiliations:** 1 Department of Psychiatry, Indiana University School of Medicine, 410 W 10th Street, Suite 2000, Indianapolis, IN 46202 USA; 2 Lighthouse Institute, Chestnut Health Systems, 221 W Walton St., Chicago, IL 60610 USA; 3 School of Social Work, Indiana University Purdue University Indianapolis, 902 West New York Street, Indianapolis, IN 46202 USA; 4 Center for Dissemination and Implementation Science, Department of Medicine, College of Medicine, University of Illinois at Chicago, 818 S. Wolcott, Chicago, IL 60612 USA; 5 Department of Psychology, Indiana University Purdue University Indianapolis, 402 N. Blackford St., Indianapolis, IN 46202 USA; 6 Center for Health Information and Communication, Health Services Research and Development, Richard L. Roudebush VAMC, 1481 W. 10th St. (11H) Rm. C8108, Indianapolis, IN 46202 USA

**Keywords:** Pragmatic trial, Opioid use disorder, Overdose, Medication for opioid use disorder, Implementation, Consolidated Framework for Implementation Research, External validity, Emergency department

## Abstract

**Background:**

This manuscript provides a research update to the ongoing pragmatic trial of Project POINT (Planned Outreach, Intervention, Naloxone, and Treatment), an emergency department-based peer recovery coaching intervention for linking patients with opioid use disorder to evidence-based treatment. The research team has encountered a number of challenges related to the “real-world” study setting since the trial began. Using an implementation science lens, we sought to identify and describe barriers impacting both the intervention and research protocols of the POINT study, which are often intertwined in pragmatic trials due to the focus on external validity.

**Method:**

Qualitative data were collected from 3 peer recovery coaches, 2 peer recovery coach supervisors, and 3 members of the research team. Questions and deductive qualitative analysis were guided by the Consolidated Framework for Implementation Research (CFIR).

**Results:**

Nine unique barriers were noted, with 5 of these barriers impacting intervention and research protocol implementation simultaneously. These simultaneous barriers were timing of intervention delivery, ineffective communication with emergency department staff, lack of privacy in the emergency department, the fast-paced emergency department setting, and patient’s limited resources. Together, these barriers represent the intervention characteristics, inner setting, and outer setting domains of the CFIR.

**Conclusion:**

Results highlight the utility of employing an implementation science framework to assess implementation issues in pragmatic trials and how this approach might be used as a quality assurance mechanism given the considerable overlap that exists between research and intervention protocols in real-world trial settings. Previously undocumented changes to the trial design that have been made as a result of the identified barriers are discussed.

## Introduction

This paper provides a research update to a previously published manuscript of a pilot randomized pragmatic trial of Project Planned Outreach, Intervention, Naloxone, and Treatment (POINT), an emergency department (ED)-based intervention for engaging patients with opioid use disorder (OUD) and connecting them to evidence-based treatment [1]. The urgent need for interventions with potential to help address the opioid crisis is reflected in the 122% increase in opioid-related overdose deaths between 2010 and 2018 in the USA [2]. This urgency has resulted in the development and implementation of a number of novel interventions that require rapid yet rigorous scientific assessment, which is not conducive to traditional explanatory trials [3]. Pragmatic trials are a novel and effective solution to meet this challenge, as they are able to deliver more immediate and generalizable solutions due to their situation in “real-world” contexts [4, 5]. The current research update is data informed in that we qualitatively documented and assessed challenges encountered since moving from the POINT pilot study to the full trial. Our approach is unique in that we use an implementation science lens to understand barriers to both research and intervention implementation, which are highly interwoven within pragmatic trials. After presenting interview findings, we provide a discussion of the impact of these barriers on the study and their implications.

### Overview of the POINT pragmatic trial

Described in full within a prior publication [1], POINT is an ED-based peer recovery coaching (PRC) intervention aimed at linking OUD patients to evidence-based treatment medications (e.g., methadone, long-acting injectable naltrexone, or one of multiple buprenorphine formulations) and additional supportive services. PRCs are paraprofessionals with lived experience of substance use disorder (SUD) recovery who provide support to individuals currently struggling with an SUD [6]. PRCs are being embedded in EDs across the USA because they are believed to be better-suited for engaging patients presenting with SUDs and motivating them to seek treatment [7, 8]. However, evidence to support this assertion regarding PRCs, whether in EDs or other environments, is currently lacking [6, 9, 10].

The POINT trial is funded by a unique mechanism that required researchers to take advantage of research opportunities presented by the US Substance Abuse and Mental Health Services Administration’s Opioid State Targeted Response grants [11], and our study leverages Indiana’s State Targeted Response-supported ED-PRC initiative to replicate and test POINT. POINT preceded and served as partial inspiration for the Indiana initiative, making it a logical intervention of research focus. A pragmatic trial design was the appropriate choice for the study due to (a) our limited ability to control all aspects of the design because of the need to work within the confines of state plans, (b) the rapid timeline with which the federal funding had to be implemented, and (c) the need for rapid translation given the gravity of the epidemic.

Pilot work for the POINT study and the protocol as finalized based on pilot results has been previously published in detail and is also available on ClinicalTrials.gov (Registration# NCT03336268) [1]. As an overview of the pragmatic trial design, the study is occurring in two Indiana-based hospitals, one urban and one serving a largely rural population. There are two arms: (1) POINT services delivered by a PRC and (2) standard care (SC) consisting of the provision of treatment referral resources by a research assistant. Each PRC and research assistant is assigned to work at a single hospital site. Cluster randomization is time-based, happening at the shift-level with the workday being divided into two shifts—8 am–3:59 pm and 4 pm–11:59 pm—with one POINT and one SC shift randomly assigned each day. Participant eligibility criteria include the following: (a) has been revived from a drug overdose or admitted to the ED for an opioid-related health issue, including opioid withdrawal, abscess (from intravenous opioid use), endocarditis (from intravenous opioid use), or active opioid intoxication; (b) scores at least “1” on the opioid use disorder screening tool; (c) be 18 or older; (d) be approved for discharge from the ED by hospital staff; and (e) and be medically stable and capable of providing consent. Staff are alerted to the presence of an eligible patient through an electronic alert or by ED staff contacting them directly. POINT arm participants are consented by a PRC who delivers POINT services and SC participants are consented by a research assistant. Baseline data are collected through a structured interview conducted at the patient’s bedside. All follow-up data is collected from existing administrative sources: the electronic health record; state systems reflecting prescription drug monitoring, child welfare involvement, and vital records; and publicly available criminal justice data.

Table 1 summarizes the extent to which pragmatism is embedded in the POINT study design using the Pragmatic Explanatory Continuum Indicator (PRECIS-2) tool [12]. PRECIS-2 recognizes that the line between pragmatic and explanatory trials is not always clear-cut and provides a mechanism for researchers to rate 9 categories of design choices along a 5-point continuum (1/very explanatory, 2/rather explanatory, 3/equally pragmatic/explanatory, 4/rather pragmatic, and 5/very pragmatic). The ratings included in Table 1 are based on the POINT design at the conclusion of the pilot: based on the two principal investigators' ratings using the tool, POINT falls largely on the pragmatic side of the continuum.

**Table 1:** PRECIS-2 ratings to demonstrate the extent of pragmatism in the trial design immediately following the pilot study

Domain	Score^a^	Rational
Eligibility criteria	4	All patients with opioid-related issues are study eligible except those who are not able to provide informed consent by the time of ED discharge.
Recruitment path	5	Patients are recruited when they are present in the ED or on an inpatient unit if they were admitted there from the ED.
Setting	4	The trial is occurring in university-affiliated teaching hospitals; however, the specific EDs do not regularly engage in research.
Organization intervention	3	The research grant provides the hospital with funding and resources to implement and run the intervention; however, the hospital employs and supervises PRCs.
Flex of experimental intervention delivery	3	PRCs are given guidelines for how to engage patients, but implementation of guidelines is not regularly monitored.
Flex of experimental intervention adherence	5	Patients are not expected to adhere to the intervention; rather, it is up to the PRCs to engage and motivate them.
Follow-up	5	All follow-up data collection is through existing administrative sources.
Outcome	5	Primary outcomes are (1) treatment linkage, (2) treatment engagement, (3) reduced overdose, and (4) reduced overdose mortality, which are all relevant to people in OUD recovery.
Analysis	5	An intention-to-treat approach is followed.

^a^1/very explanatory, 2/rather explanatory, 3/equally pragmatic/explanatory, 4/rather pragmatic, and 5/very pragmatic

While our 6-month pilot was conducted with relatively minor difficulties and successfully met its goals [1], the study and the hospitals it is set within are dynamic entities, and new challenges have been encountered with the addition of a new site, onboarding of new personnel, and changes in the lineup of key stakeholders who support our work. As such, we have encountered a number of challenges since embarking on the full trial. Most notably, we are far from reaching our target enrollment of 712. As of March 21, 2020, when we had to stop recruitment due to the coronavirus disease 2019 pandemic, we had enrolled 238 subjects (*n* = 150 POINT; *n* = 88 SC) and experienced 52% lower enrollment in the SC arm. In July 2020 when this manuscript was originally drafted, the trial was more than halfway complete, making it unlikely we would meet our enrollment target in the time remaining. Given the distinctive nature of the project coupled with the importance of partnered, pragmatic trials, we sought to understand the issues facing the trial using an implementation science approach.

### Using implementation science to understand research-intervention intersections

Pragmatic trials and implementation research share a common goal in attempting to make healthcare research more translatable to real-world practice [13]; however, the means to this end differ between the two. Whereas pragmatic trials focus on making research conditions as similar as possible to the actual circumstances under which the intervention will eventually be delivered, the field of implementation science has largely (though not explicitly) been concerned with identifying strategies for moving research from highly controlled explanatory trials into practice. Both of these pursuits require a rich understanding of the context in which a particular intervention of focus has been, is, or will be delivered [13–15]. While there are no specified approaches for documenting context in a pragmatic trial, implementation scientists have developed a number of tools to assess implementation determinants that can be systematically applied to document barriers and facilitators to intervention implementation. While these tools can be used within a pragmatic trial, they can also be broadened beyond their traditional focus on aspects of intervention implementation to include implementation of research procedures. This is because the attention to external validity within a pragmatic trial means there is often a high degree of overlap between procedures guiding the intervention and the research that should both be isolated and understood to improve subsequent translation.

We employed the Consolidated Framework for Implementation Research (CFIR) as the guiding framework for our qualitative inquiry of challenges impacting the POINT trial. We selected the CFIR as the guiding implementation framework because it was developed to be applied specifically within a clinical context and offers a highly detailed list of domains and constructs that lend themselves well to deductive qualitative work [16–18]. The CFIR comprises 37 implementation determinant constructs organized in 5 domains: inner setting, outer setting, intervention characteristics, characteristics of individuals, and the implementation process. While the CFIR has been used to investigate intervention implementation within at least one pragmatic trial [19], to our knowledge, it has not been used to investigate research protocol implementation.

## Method

A rapid qualitative investigation was undertaken with the goal of identifying implementation barriers affecting the POINT intervention and research protocols, as well as the areas of intersection between the two. Rapid qualitative research has been demonstrated to be a beneficial approach for implementation studies facing time and resource constraints [20]. In this specific case, there was a need to complete data collection and analysis quickly to provide actionable feedback before the trial’s end. Time constraints impacted additional choices such as focusing on barriers (as opposed to barriers and facilitators) within the analysis and who would be interviewed given their knowledge of the study and availability. All data collection and analysis were led by AD (the first author). The principal investigators requested AD conduct this inquiry due to a need to understand factors impacting the study that could guide course corrections and because she had no prior engagement with the POINT study, meaning her findings would not be affected by prior experience with the trial.

A total of 4 interviews were conducted with individuals involved in the study: a group interview with three research personnel (e.g., the two principal investigators and the project manager), a group interview with two of the three PRCs working at the urban hospital, an individual interview with one of the two PRCs working at the rural hospital (this PRC had had recently left the study for a new position but had the most seniority at this location), and one group interview with two PRC supervisors at the rural location who provided clinical and administrative supervision to study PRCs but who were not involved in the research protocol or data collection (the PRC supervisor at the urban location had recently left their position and was unable to be reached for an interview). Due to the rapid nature of the study, interviews were scheduled so they could be completed as quickly as possible based on participants’ availability, and two PRCs (one at each hospital) were not interviewed due to work and personal conflicts in their schedules. All interview questions were adapted from a qualitative interview guide tool found at cfirguid.org [21]. First, relevant constructs were chosen across the 5 CFIR domains. Question prompts were then tailored to fit the study context (e.g., inner setting infrastructure’s impact on implementation was tailored to reflect how the infrastructure of the ED affected the implementation of the study protocol as well as the implementation of the PRC intervention). Interview guides for PRCs, supervisors, and investigators were similar; however, all interviews were semi-structured, allowing the researcher to pursue unexpected lines of inquiry. Interviews lasted 30–60 min and were audio recorded and transcribed prior to analysis.

Deductive analysis of the data was conducted in MAXQDA qualitative data analysis software [22]. AD conducted an initial wave of coding using a guide based on the CFIR domains, identifying only barrier-focused determinants [23]. For each CFIR-coded passage, she then applied a second-level code indicating whether the specific barrier mentioned was related to the implementation of the (a) intervention or (b) research protocols, allowing for double coding to indicate areas where barriers were related to (c) both intervention and research protocols. Following this, more focused coding of content into specific constructs within the CFIR domains was conducted with the assistance of a second coder (MZ). Throughout this process, the coders met to discuss and further refine themes. The initial average intercoder reliability (calculated as percent agreement across four transcripts) was 85.3%. Intercoder reliability for the first round of coding was 100% across PRC interviews, 75% for the researcher interview, and 60% for the supervisor interview. Discrepancies were discussed between coders and revised until 100% agreement was met. Findings from the analysis presented below focus on barriers identified to be relevant/significant across sites or that had a significant impact on implementation (e.g., common barriers across interviews and/or barriers mentioned multiple times within an interview). Of note, not all barriers presented were identified across all interviews, as we found hospital workers (e.g., PRCs and supervisors) and researcher staff perceived/experienced different issues in research and intervention implementation.

## Results

Findings from the analysis are summarized in Table 2, which lists the barriers identified through the analysis process, providing: (a) a description of the barrier as it pertains to its situation (e.g., intervention, research, or both), (b) the CFIR domain and construct the barrier corresponds to; (c) the specific barrier/issue as discussed in the interview, (d) the source (e.g., researchers, PRCs, or PRC supervisors), and (e) an example quote. We describe each of the barriers with more detail in the sections that follow.

**Table 2:** Barriers identified through interviews

Situation of barrier/challenge	CFIR construct (domain)	Specific barriers/issues discussed	Source	Example quote
Intervention	Complexity (study characteristics^a^)	Complexity of research protocol	PRCs	*… having them [patients] sit there and be patient through all these questions [before providing services] … they are starting to get annoyed.* (PRC)
	Culture (inner setting)	ED staff lack SUD knowledge	PRCs, supervisors, researchers	*[ED staff] were not well educated on how to work with opioid use disorder … we have actually had physicians come to our coaches asking how they should treat a patient.* (Researcher)
	Patient needs and resources (outer setting)	Lack of community referral resources	PRCs, supervisors, researchers	*… there’s still just a disconnect between the resources [in the community], and it takes a long time to get someone in somewhere …* (PRC)
Research	Personal attributes (characteristic of individuals)	PRCs are better at study recruitment	PRCs, supervisors, researchers	*… the research assistants were not … being visible in that area [the ED] as what they needed to be”* (supervisor)
Intervention and research	Complexity (intervention characteristics)	Timing of intervention delivery	PRCs, supervisors	*… it’s inherently difficult [to speak with patients] … you deal with people who were again, highly sedated, often times.* (PRC)
	Networks and communication (inner setting)	Ineffective communication with ED staff	PRCs, supervisors, researchers	*… [nurses] are so busy and there is so much information coming at them … there is no time for meetings throughout the day {to get] in front of them for a half hour and kind of explain what we do and who is appropriate [for the study].* (PRC)
	Structure (inner setting)	ED lacks privacy	PRCs, supervisors	*… a lot of our patients are in the hallway … they say the entire ED is a private area but when you are talking to someone … being asked questions about their drug use … can be a little off-putting for some people.* (PRC)
	Culture (inner setting)	Fast-paced ED setting	PRCs, supervisors, researchers	… *to get [patients] to do the study itself … they had to have a discharge order, and so you had a very tight window to get in there and engage …*. (PRC)
	Patient needs and resources (outer setting)	Patients’ limited resources	PRCs, researchers	*… I’d have that initial intervention there in the ED and then a huge barrier was follow-up … a lot of them did not have working phones, you know there was no family support that you could contact* (PRC)

^a^“Study characteristics” is not a CFIR domain. As such, this is a parallel to CFIR “intervention characteristics” domain

### Barriers unique to intervention implementation

#### Complexity of research protocol (study characteristics)

PRCs and their supervisors discussed how complex characteristics of the *research* protocol (a parallel to CFIR’s intervention characteristics domain) interfered with PRC’s implementation of intervention procedures. For example, the length of recruitment and data collection protocols prevented PRCs from effectively engaging with patients, which is one of their primary functions. PRCs described the protocols as both “lengthy” and “tedious” for patients due to the amount of information that has to be reviewed: “… having them [patients] sit there and be patient through all these questions … I can see in their body language they are irritable, restless, and they’re starting to get annoyed.” Relatedly, reflecting on data collection, a PRC supervisor stated: “… they [patients] may be perfectly happy to get the resources [PRCs offer to them] and all of those things, but when it comes down to interviewing [them] … they [the patients] kind of go, ‘ah, no’.” This highlights that despite patients’ receptivity to the intervention (i.e., interest in PRC services), study procedures often stymied PRC’s work engaging them. Speaking directly to the issue of engagement, one PRC stated: “reading these disclosures [for the consent process] and everything else … it could be perceived that you were losing that kind of human touch … like you were recruiting them for something you needed.” From this PRC’s perspective, the consent process made it difficult to demonstrate empathy and establish a genuine rapport with patients as is a primary goal of PRC-patient interactions. While these issues likely also had impacts on research implementation, this was only framed as an intervention barrier in interviews.

#### Culture (inner setting)

PRCs, their supervisors, and researchers discussed how the ED’s lack of training and specialization in treating patients with SUD, particularly OUD, affected intervention implementation. For example, both PRCs and researchers shared multiple instances when ED staff, such as physicians, consulted PRCs for guidance regarding treatment of withdrawal symptoms or medications for OUD (of note, PRCs were instructed that they could not provide medical advice and were given a list of online resources with evidence-based information to provide physicians instead, and the principal investigators also shared this information with hospital leadership) . This resulted in what was perceived by researchers as lack of knowledge regarding and resistance to current evidence-based practices for treating OUD. Most importantly, the ED was not using buprenorphine (a type of OUD medication) bridging, which is an evidence-based practice that improves treatment linkage by inducing patients on buprenorphine in order to prevent withdrawal symptoms until they can be seen by a long-term treatment provider, and this practice has faced barriers to implementation across the USA where there is currently no ED national standard of care for OUD treatment linkage (see [24]).

buprenorphine bridging would make this intervention [POINT] so much more effective … one of the biggest barriers [PRCs] have is someone getting sent out the door and they’re going to start detoxing and [as a result] … they don’t make it to the [treatment] appointment they set up for them in the next 24 to 72 hours. (Researcher interview)

PRCs also noted that ED staff’s lack of knowledge and training in treating SUD generally and OUD specifically resulted in stigma toward OUD patients and made PRCs’ jobs more difficult. One PRC explained how some ED staff who do not have an understanding of the chronic course of SUDs do not feel patients really want to work toward addressing their OUD because “this person was in here last week,” as if their return visits to the ED made these patients undeserving of services. This in turn made it more difficult for PRCs to do their job because ED staff were more focused on quickly discharging these patients, making PRC’s work more difficult.

#### Patient needs and resources (outer setting)

All three types of interviewees discussed how lack of community-based resources affected intervention implementation. PRCs are to assist individuals in connecting to treatment and supportive services. However, the lack of availability of appropriate resources in the community made this challenging. For example, many local treatment centers or sober living facilities did not accept individuals receiving medications for the treatment of OUD. One PRC explained this as a “disconnect” between the local resources available and evidence-based treatments for OUD, which presented a particular barrier to working with OUD patients experiencing homelessness. Researchers also highlighted the deficit in local providers and organizations that support OUD treatment medications. PRCs, researchers, and supervisors stressed the importance of having an affiliation or close collaborative relationship with treatment facilities or clinics that can prescribe medications to treat OUD to ensure efficient and effective transition to care but that these relationships were difficult to establish.

### Barrier unique to research implementation

#### Personal attributes (characteristics of individuals)

All three categories of interviewees discussed how the SC arm’s lower enrollment rate was likely due to certain advantages PRCs had over research assistants. First, as hospital employees, PRCs had a duty to serve other SUD patients in the ED. As a result, PRCs were present in the ED more often and able to form stronger relationships with ED staff, which resulted in more opportunities for PRCs to be informed of study-eligible patients. As one researcher explained, “[coaches] have the ability to both recognize patients and engage patients earlier that our RAs [research assistants] don’t have, and we’ve tried to level that playing field as much as possible [through more stringent engagement procedures] but it’s difficult.” PRCs’ status as hospital employees resulted in additional difficulties following the study protocol:

… no matter how much you tell these recovery coaches that they need to follow study procedures, it’s really hard for them to be able to do that because...in the moment, they have to make decisions, one, and they can’t just deny seeing a patient if a nurse pulls them into a room, saying this patient needs help because we have our study procedures that they have to go through. (Researcher interview)

Supervisors also highlighted differences in PRC versus research assistant interactions with ED staff and attempts to improve this: “… the research assistants were not [as] visible in that area as what they needed to be … [researchers need to] try and get them to be more engag [ed].”

Lastly, supervisors and researchers noted frequent turnover of PRCs made it challenging to maintain steady study progress: as one supervisor put it, “… it takes time to get them hired, it takes time to get them oriented and trained.” At the time of the interviews, four PRCs at the urban hospital and three at the rural hospital had resigned or been terminated. Researchers and supervisors highlighted a number of reasons related to staff turnover such as “incompatibilities of the coaches and the intervention” related to PRC’s personal biases against OUD treatment medications, “certain coaches not feeling comfortable working with opioid use disorder” as opposed to other SUDs, and SUD relapse (i.e., a return to active substance use) among PRCs. Another researcher explained how some “coaches don’t follow protocols and procedures and we’ve had to let them go” when the issue was identified and could not be solved with corrective actions. Yet another reason for turnover was PRCs accepting new jobs. While researchers acknowledged this was a positive outcome in cases where the job paid more or was more compatible with the PRC’s life situation, it was also frustrating because “… when you have a really good person, they go somewhere else … so that has been difficult for us.”

### Barriers to both intervention and research implementation

#### Complexity (intervention characteristics)

PRCs and their supervisors highlighted how the time at which the intervention is delivered resulted in challenges. Most importantly, a key aspect of the intervention is utilizing a time of crisis as a critical moment when the patient might be receptive to entering treatment. However, there were challenges engaging patients who were in an altered physical, emotional, and/or mental state in the ED. As described by one PRC “people coming in are overdosed, are on Narcan [an overdose reversal drug that precipitates rapid withdrawal symptoms] … or they’re just not lucid enough to kind of truly engage ….” One supervisor voiced similar concerns “… they are not in the right mental health state moment to kind of hear and sign up for that.” From a research perspective, this means PRCs often had to make multiple engagement attempts until the patient demonstrated they were capable of providing informed consent, and a patient could be discharged from the ED between these attempts resulting in a missed opportunity for enrollment. From an intervention perspective, the high competency consent threshold prevented PRCs from effectively engaging with patients as they would outside of the research context.

#### Networks and communication (inner setting)

According to PRCs, their supervisors, and researchers, the inability to effectively network and establish lines of communication with ED staff was a major barrier. One PRC noted the challenge in connecting with ED staff:

… they’re so busy and there is so much information that is coming at them … I again understand that that is the nature of nursing, is there is no time for meetings throughout the day and what not … It [forming relationships] was kind of like nurse by nurse, it was a slow process of getting the nurses to understand exactly what we were doing … .

Supervisors explained that PRCs spent a lot of time in the beginning of the study making themselves known, explaining their role to ED staff, and working to build relationships with staff. However, high numbers of staff working in the ED, turnover between shifts, and medical residents rotating through the ED meant any gains in educating staff did not last long. As one PRC noted: “there’s such a change all the time and the students, the [nurses], the doctors that we have to reintroduce what it is we do … and what recovery coaches are and do, and what they are not.” It was noted by both PRCs and supervisors that communication barriers also affected study recruitment, as it took time to develop effective communication between ED staff and study personnel (e.g., PRCs and research assistants) so that the study as appropriately alerted when eligible patients presented to the ED.

#### Structure (inner setting)

PRCs and their supervisors discussed minimal privacy in the ED as a barrier to both research and intervention implementation. As one PRC explained how space limitations made it difficult to engage patients: “… the entire ED is [technically] a private area but when … someone who’s there because they have an impacted wisdom tooth … is close by to a person who’s being asked questions about their drug use … it can be a little off-putting.” What this PRC is speaking to is the discomfort some patients might have agreeing to discuss their substance use (whether in the context of answering research or clinical questions) when there is often no more than a curtain separating them from other patients.

#### Culture (inner setting)

All three interviewee types noted that the study protocol and intervention were incompatible with the fast-paced nature of the ED, which resulted in challenges to implementation. As one researcher explained, “the culture is shaped around the fact that their [ED staff] performance is based on how fast they get people in and out of the ED ….” However, PRCs and research assistants could not complete study procedures until an individual was medically cleared for discharge (a signal to research staff that they are likely competent to provide informed consent), thus extending the time patients were in the ED. This was also an inconvenience for patients, as they were regularly eager to leave the ED by the time they were medically cleared, and they often did not want to stay to complete an interview or enroll in services.

#### Patient needs and resources (outer setting)

PRCs identified how characteristics of the population created barriers to study and intervention implementation. They stated many participants had limited resources (e.g., insurance, stable housing, a stable phone number to be reached at), which made it difficult for PRCs to follow-up with them after they left the ED. As one PRC explained:

[Patients] lives are completely in flux and unstable … a huge barrier was follow-up … a lot of them didn’t have working phones, there was no family support that you could contact, so a lot of times you’d have a good initial interaction … but given the demographic of the population we served it was often tough to get that follow-up.

This was primarily an intervention-related barrier since the study followed participants through administrative data. However, researchers were seeking to recruit a small number of participants for follow-up qualitative interviews related to their recovery experiences after leaving the ED, and the inability to locate them has interfered with timely completion of this research aim of the main trial.

## Discussion

The lack of strict controls within pragmatic trials means researchers must document and report extensive contextual information to ensure their results are interpretable, useful to future practice, and replicable [13]. We identified nine unique barriers impacting the POINT study through analysis of interviews with PRCs, their supervisors, and members of the research team. Three of these barriers specifically impacted intervention implementation, one impacted research implementation, and five impacted both the intervention and research implementation. The fact that the majority of barriers cut across aspects of both intervention and research implementation *demonstrates the high degree to which aspects of the intervention of focus and research protocols can be intertwined within a pragmatic trial*. The tension between real-world service delivery and pragmatic trial protocols has been discussed in prior literature [12, 19, 25, 26]; however, these discussions have largely focused on how attention to external validity can lead to questionable implementation of the intervention and type III error (i.e., the attribution of outcomes to an intervention that was never fully implemented) [4, 19, 27, 28]. Our findings demonstrate how the real-world research context can impact full implementation of both intervention and research protocols, even when protocols have been developed to accommodate the local service setting (see Table 1). In the rest of this discussion, we focus specifically on those barriers that were identified as concurrently impacting research and implementation.

Complexity of interventions is a previously noted barrier for pragmatic trials, as they often have several interacting components that depend on skills and interactions among many providers [5, 29]. This complexity reinforces the need for pragmatic trial research protocols to be as unobtrusive as possible to ensure they do not get in the way of intervention delivery. This is best demonstrated within the POINT study by time at which patients are identified and engaged by coaches, as success required interaction to occur at a particular and fleeting point when they are receptive to intervention, capable of providing research consent, and willing to sit through a structured research interview. It can be particularly frustrating for PRCs to wait for patients to be ready to provide research consent before they begin delivering services, as this would not be the case outside of the study. As such, the real issue at play here are the informed consent procedures, and the pragmatic trial literature does suggest simplifying or waiving consent in situations where it can lead to impracticability of research [5, 30]. While the lead researchers did seek to obtain a waiver of informed consent because of impracticability related to the urgency of ED-based care, they ultimately decided not to pursue this upon consultation received from staff in the university’s human subjects compliance office. The incongruity between clinical and research standards for informed consent raises important ethical issues. While the research standard for informed consent must take into account that research may not directly benefit the participant, and therefore it is reasonable to hold a higher standard, it is also worth asking the degree to which the clinical standard is lowered in some settings is for the direct benefit of the patient (e.g., urgent interventions may be necessary to revive the patient to the point of being able to consent) versus for the benefit of the healthcare system (e.g., intervening before full consent can be obtained in order to decrease discharge times) [31]. If a waiver of informed consent is requested in pragmatic trials, heightened data security measures such as using a data broker who provides limited or de-identified datasets should be employed to protect against risks related to a loss of confidentiality.

The inner setting where the intervention is delivered is a frequently documented barrier in implementation science and pragmatic trials [19, 32], and aspects of the ED’s communication networks, culture, and structure were demonstrated barriers for POINT intervention and research protocols. These issues were apparent at both hospitals, but, while not captured in the interviews, they were much more difficult to address in the urban hospital site where frequent changes in leadership led to less support for the study over time. This was compounded by the loss of one of POINT’s collaborators, an ED physician who was acting as primary liaison between the study and the ED. As sufficient organizational support is critical to pragmatic trial success [5], the research team has tried several routes to boost the relationships between the study and the ED with limited success. The implementation literature points to several strategies for garnering organizational support that can be looked to for additional strategies such as engaging stakeholders “early and often” and leveraging local champions and informal leaders [33].

The outer setting is often not well described in implementation or pragmatic trial literature due to considerable focus that is paid to the inner setting. However, the outer setting can have significant impacts for complex interventions like POINT that reach beyond the boundaries of the immediate delivery setting [34, 35]. POINT specifically is dependent on relationships with prescribers to whom PRCs can refer patients, and even PRCs who excel at engaging patients cannot impact OUD outcomes without these relationships. While the original POINT program had a dedicated clinic to prescribe medications with walk-in hours specifically for POINT patients, there were no such affiliated clinics at either of the hospital sites. Because of this, the lead researchers played an active role to help PRCs establish relationships with outside providers and develop referral protocols to connect POINT patients with treatment. This raises an external validity issue, as the researchers’ networks would not have been available to help establish these relationships outside of the study’s context.

### Limitations

Regarding limitations, the need for rapid inquiry prevented interviews with research assistants and ED staff. ED staff in particular have busy schedules that, over the course of the project, have required significant numbers of attempts and lead time to schedule interactions on the part of the research team. However, one of the PRC supervisors interviewed was a member of the ED staff, and the PRCs and researchers do have a decent understanding of the perspectives of both groups given their frequent interactions with research assistants and multiple meetings with ED leaders and physicians that had been held regarding study implementation. The use of both group and individual interviews is another limitation, as the types of information participants are willing to share might differ by modality. Due to the study goals and timeline, the analysis was aimed at identifying barriers that had led to poor performance in an effort to identify areas for improvement before the study’s end. However, the CFIR is intended to examine both facilitators and barriers as implementation determinants, and future work in this area could benefit from investigating intervention and research protocol facilitators within pragmatic trials. Finally, the approach employed was limited in its late-stage and cross-sectional nature, and it could potentially be used to guide quality assurance and strategic decision-making if performed early and often enough.

### Changes to the trial design moving forward

Potential solutions to the POINT trial’s barriers discussed above would include such actions as going back to the IRB to argue for a waiver of informed consent based on demonstrated impracticability and identifying and implementing proven strategies for engaging ED staff and external providers. However, since the interviews were completed, the study has faced an even greater challenge related to the coronavirus pandemic that has taken priority. While PRCs were allowed to continue delivering services in the hospital, data collection was stopped in late March 2020 due to the need to ensure staff safety and the suspending of research at the study’s home institution. There has also been additional PRC turnover in the urban hospital site during this time, resulting in a need to hire and train new PRCs before the study can resume. Given these setbacks, it will take considerable time to restart activities at this location. With the study entering its last year, the lead researchers have decided, in consultation with the study’s funder, to stop enrollment at the urban site and focus attention on the rural location. In addition, the study will utilize the electronic health record to identify study-eligible patients who entered the ED during times when the study was running and assign them to the appropriate arm based on the time they were discharged, resulting in an intent-to-treat design that recognizes not all patients presenting to the ED when a PRC was working actually engaged POINT. Health record data will be combined with existing state data and provided to the researchers in a limited dataset. While no more original data will be collected at the urban site moving forward, this change will potentially double the sample size of the study by retrospectively identifying patients perviously not identified and/or engaged by PRCs or research assistants. Researchers still plan to use baseline data already collected to conduct exploratory analyses to identify latent subgroups of patients and the extent to which they might differ in responsiveness to the intervention.

## Conclusion

Our experience points to the utility of using an implementation science lens, broadly, and the CFIR framework specifically, to examine not only the factors affecting the intervention implementation but also the research protocol. Such analyses allow researchers to understand the independent and concurrent effects of contextual factors. Perhaps of equal importance, our inquiry highlights the extent to which intervention and pragmatic trial naturally mutually adapt to one another. While this phenomenon may be nettlesome to the pursuit of pure scientific knowledge, it should be acknowledged more regularly and beyond the mere confines of purported pragmatic trials. Results also highlight the need for future research to document the extent to which the research and intervention protocols and determinants of implementation interact and co-adapt within a trial. Given this, pragmatic trials would benefit from documentation of both barriers and facilitators, as well as how intervention and research protocols evolve in reaction to them. Such lines of inquiry could ultimately have positive impacts on practice by identifying ways to implement pragmatic trial protocols in a manner that is less intrusive to real-world clinical settings.

## Data Availability

Qualitative data are not available due to confidentiality concerns related to such a small sample.

## Abbreviations

POINT: Planned Outreach, Intervention, Naloxone, and Treatment
CFIR: Consolidated Framework for Implementation Research
ED: Emergency department
OUD: Opioid use disorder
PRC: Peer recovery coach
PRECIS-2: Pragmatic Explanatory Continuum Indicator
SC: Standard care
SUD: Substance use disorder
